# Microbial Secondary Metabolism and Biotechnology

**DOI:** 10.3390/microorganisms10010123

**Published:** 2022-01-07

**Authors:** Mireille Fouillaud, Laurent Dufossé

**Affiliations:** 1 Laboratoire de Chimie et de Biotechnologie des Produits Naturels—CHEMBIOPRO, Université de la Réunion, 15 Avenue René Cassin, CS 92003, F-97744 Saint-Denis, Ile de la Réunion, France; laurent.dufosse@univ-reunion.fr; 2 Ecole Supérieure d’Ingénieurs Réunion Océan Indien—ESIROI Agroalimentaire, 2 Rue Joseph Wetzell, F-97490 Sainte-Clotilde, Ile de la Réunion, France

**Keywords:** secondary metabolites, microorganisms, biotechnology, screening, production, extraction, bioactive properties, perspectives

## Abstract

In recent decades scientific research has demonstrated that the microbial world is infinitely richer and more surprising than we could have imagined. Every day, new molecules produced by microorganisms are discovered, and their incredible diversity has not yet delivered all of its messages. The current challenge of research is to select from the wide variety of characterized microorganisms and compounds, those which could provide rapid answers to crucial questions about human or animal health or more generally relating to society’s demands for medicine, pharmacology, nutrition or everyday well-being.

## 1. Microorganisms and Metabolites: An Incredible World of Novelty for Biotechnologists, New Opportunities for Industries

Microbial secondary metabolites, now named as specialized metabolites, often have unusual structures and many have demonstrated major effects on the health, nutrition and economics of our society [1]. These compounds are usually low molecular mass products of secondary metabolism, which take place out of step with the main microbial growth phase. They include antibiotics, pigments, toxins, enzyme inhibitors, immunomodulators, effectors of ecological competition or symbiosis, or compounds with hormonal activity or particular effects on lipids or carbohydrates metabolism. Some have already established themselves as antimicrobials, antivirals, antioxidants, antitumorals, vaso-relaxants or contractants, diuretics or laxatives. Others are used as colorants, pesticides, or growth promoters for animals or plants [2]. Approximately 53% of the FDA-approved natural products-based drugs are originating from microorganisms [3].

The synthesis of specialized metabolites is finely adjusted by nutritive sources, growth conditions, feedback control, enzyme induction or inactivation. Their regulation is often influenced by specific low molecular mass compounds, also transfer RNA, σ factors and gene products formed during post-exponential development. Recent research demonstrated that the production of specialized metabolites is mostly coded by clustered genes on chromosomal DNA rather than by plasmidic DNA. However, the related pathways are still not fully clarified and thus provide a new theoretical frontier for academic researchers in enzymology, control and differentiation [4]. 

Omic sciences such as genomic, transcriptomic or metabolomic applied to industrial microorganisms now offer new opportunities for strain discovery characterization and improvement. Thus, great potential exists for the development of novel compounds for pharmaceutical, nutraceutical, dyeing, or agricultural industries.

**Figure 1:**
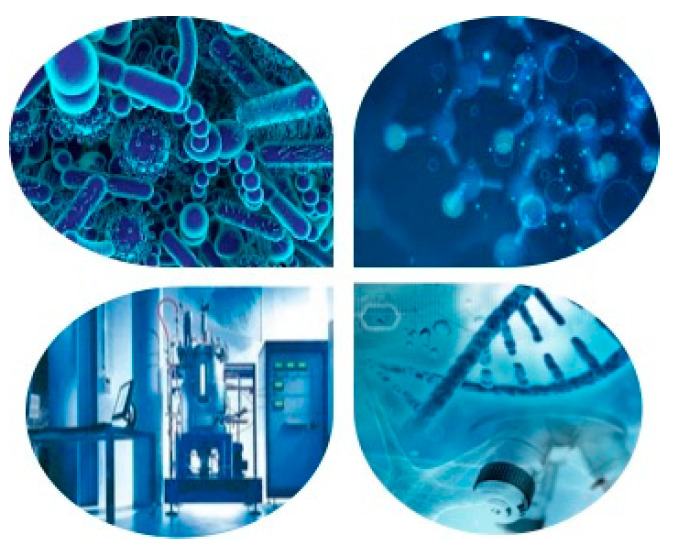
Illustration of the diversity of scientific fields underlying microbial biotechnology (clockwise): microbiology, chemistry, genomic and industrial technologies.

In this special issue secondary metabolites produced by bacteria, actinomycetes, fungi or microalgae are investigated under different facets linked with bioactivity or other interesting properties. Biotechnology combines a range of scientific fields that connect basic and applied research (Figure 1). This is the initial but fundamental step towards the resolution of technical problems, anticipating industrial exploitation.

The era of microbial natural products manufactured through biotechnology has just begun.

## 2. From the Beginning: Screening and Characterization of Valuable Strains

Not so long ago, scientists discovered that the marine realm is an incredibly rich biotope for the discovery of new microorganisms and subsequently the characterization of valuable new molecules. 

Sponges and their associated microbiota have been found to produce a wide variety of bioactive secondary metabolites. In the pharmaceutical field, several natural products extracted from marine organisms have already demonstrated their capacity to delay aging and/or extend lifespan. However, the biodiversity from the Southwest of the Indian Ocean is much less studied, especially regarding anti-aging activities. In the study presented by Saïd-Hassane et al. (2020) [5], the microbial diversity of the marine sponge *Scopalina hapalia* was investigated by metagenomic analysis. Twenty-six bacterial and two archaeal phyla were recovered from the sponge, of which the Proteobacteria phylum was the most abundant. Thirty isolates affiliated to the genera *Bacillus*, *Micromonospora*, *Rhodoccocus*, *Salinispora*, *Aspergillus*, *Chaetomium*, *Nigrospora* and or related to the family Thermoactinomycetaceae were cultivated for secondary metabolites production. Crude extracts from selected microbial cultures were found to be active against elastase, tyrosinase, catalase, sirtuin 1, CDK7 (Cyclin-dependent kinase 7), Fyn kinase and proteasome. These results highlight the potential of microorganisms associated with a marine sponge from the Indian Ocean to produce anti-aging compounds.

Marine epiphytic bacteria are also highly diverse, and ubiquitous in marine biotopes where they have to survive constant pressures coming from physicochemical parameters (hydrostatic pressure, low oxygenation, salinity…), biotic competition or predation (e.g., protozoans and nematodes). These marine organisms have developed defense strategies to face adversity, for example by producing toxic bioactive compounds. Several active metabolites have been identified from these microorganisms coming from specific genes expression. The review of Salikin et al. (2020) [6] focuses on the potential of marine epiphytic bacteria to be a new platform for novel anti-nematode drug development. Emerging strategies, including culture-independent high-throughput screening to discover new strains are highlighted.

The review by Nawaz et al. (2020) [7] focuses on pigments and provides an overview of marine bacteria synthetizing bio-pigments, along with their applications. It highlights a range of molecules already valued at the industrial level such as prodigiosin, astaxanthin, violacein, zeaxanthin, lutein or lycopene.

Insect-associated bacteria, are supposed to be involved in the life cycle of their host due to the panel of secondary metabolites they are able to produce. These strains are certainly one of the less explored sources of new active compounds. With the aim of efficiently discovering new bioactive molecules, the diversification of the culture conditions of one strain may induce the activation of diverse biosynthetic gene clusters. This OSMAC approach (one strain many compounds) is based on the fact that some microbial metabolites are not produced under certain set of physicochemical parameters and may appear when the conditions are modified. Inspired by these two approaches, the production of the cyclic pentapeptides pentaminomycins C, D and E was significantly improved in the culture of the *Streptomyces* sp. GG23 strain, an actinobacteria isolated from the guts of *Tenebrio molitor* (the mealworm beetle) [8]. The analysis of the non-ribosomal peptide biosynthetic gene cluster suggested that the unicity of the compounds, based on the structural variations, originates from the low specificity of the adenylation domain in the non-ribosomal peptide synthetase (NRPS) module 1, indicating that macrocyclization can be catalyzed non-canonically by penicillin binding protein (PBP)-type TE. Additionally, pentaminomycins C and D exhibited significant autophagy-inducing activities and were cytoprotective against menadione-induced oxidative stress in vitro. 

One original domain of exploration is the analysis of secondary metabolites obtained from microbial co-cultures, via metabolome tools. Among the rich microbiote isolated from the Red Sea sponge *Coscinoderma mathewsi* (23 isolates), three actinomycetes strains were defined as novel species of the genera *Micromonospora*, *Nocardia*, and *Gordonia* [9]. This study of Shamikh et al. (2020) [9] demonstrated that *Micromonospora* sp. UA17 co-cultured with two mycolic acid-containing actinomycetes namely *Gordonia* sp. UA19 and *Nocardia* sp. UA 23, or supplemented with pure mycolic acid could produce metabolites such as a chlorocardicin, neocopiamycin A, and chicamycin B that were not found in the respective monocultures. This implies a mycolic acid effect on the induction of cryptic natural product biosynthetic pathways and reveals that silent biosynthetic gene clusters can show their unusual capacities for the production of secondary metabolites, under unclassical cultivation conditions. 

Siderophores, as serratiochelins, are specialized compounds with high affinity for ferric iron, that are produced by the opportunistic pathogen *Serratia marcescens.* The siderophores are of pharmaceutical interest as they can be used in their native form to treat iron overload diseases or facilitate uptake of antibiotics by bacteria, through siderophore-antibiotic drug conjugate. In the study of Schneider (2020) [10], the rare siderophore serratiochelin A was extracted with high yields from an iron-depleted co-culture of *Serratia* sp. and *Shewanella* sp. (a strain from marine environment also involved in iron cycle). As this molecule was not observed in axenic cultures of *Shewanella* or *Serratia*, the co-culturing may induce the production of the compound, possibly because of the competition for iron between the two strains in the culture medium. The serratiochelin A antibacterial effect was tested and was specific towards *S. aureus.* The molecule also exhibited toxic effects on both eukaryotic cell lines A2058 and MRC5. 

Thereafter, the construction of microbe consortia opens up an endless avenue of exploration towards the production of new bioactive molecules.

## 3. New Molecules

*Streptomyces* is a very studied genus, historically known for the production of bioactive compounds. However, strains of unusual origins are able to furnish new natural products. As an example, the *Streptomyces* sp. strain IB201691-2A from the Lake Baikal endemic mollusk *Benedictia baicalensis* synthesizes three new angucyclines (baikalomycins A–C), as well as large quantities of rabelomycin and 5-hydroxy-rabelomycin [11]. Baikalomycins A–C exhibited varying levels of anticancer properties. Rabelomycin and 5-hydroxy-rabelomycin also showed antiproliferative activities. The gene cluster for baikalomycins biosynthesis was identified, cloned and expressed in *S. albus* J1074. Heterologous expression and deletion experiments allowed the glycosyltransferase functions implicated in the synthesis of these original compounds to be specified.

The crude extract from a culture of *Curvularia papendorfii,* an endophytic fungus isolated from *Vernonia amygdalina*, a medicinal plant from Sudan, revealed an important antiviral effect against the human coronavirus HCoV 229E and the feline coronavirus FCV F9 [12]. Additionally, a selective antibacterial activity against *Staphylococcus* sp. was observed, as well as an interesting antiproliferative competence against the human breast carcinoma MCF7 cell line. Twenty-two metabolites were identified from this extract and two major pure compounds were characterized including a new polyhydroxyacid: kheiric acid. Kheiric acid showed effective inhibition capacities against methicillin-resistant *Staphylococcus aureus* (MRSA). Hence, endophytes merit more attention, as a treasure trove of new bioactive compounds.

## 4. Increasing Knowledge on Bioactive Properties

Given the societal implications, the search for bioactive properties of bio-based molecules mobilizes considerable energy among research teams. 

The marine microalgae *Aurantiochytrium* sp. is considered a promising source for docosahexaenoic acid (DHA) production. DHA is a n-3 long-chain polyunsaturated fatty acid and is critical for cellular processes involved in maintaining health. In the study of Liu et al. (2020) [13], UV mutagenesis was utilized to obtain competitive *Aurantiochytrium* sp. strain with enhanced DHA production (1.90-fold higher than wild strain). The key genes related to the increasing DHA accumulation were explored by comparing the transcriptome between the mutant and the parent strain. The mRNA expression levels of *CoAT*, *AT*, *ER*, *DH*, and *MT* genes, are linked to the increased intercellular production of DHA, and can be manipulated to control DHA yields in *Aurantiochytrium* sp. The genetic improvement of microbial strains is thus only at the beginning of its immense possibilities.

Biofilms, composed of microbial secreted exopolysaccharides, protects bacteria against adverse environmental conditions but favor the contamination of surfaces as diverse as foodstuffs or medical material. A classical approach to eliminate biofilms is to use natural anti-microbial compounds. However, new chemical synergies are still emerging, as developed by Argüelles et al. (2020) [14], such as the joint use of carnosic acid (obtained from rosemary) and propolis (from honeybees’ panels), to control the pathogenic yeast *Candida albicans*. Recent advances in biofilm eradication also involve the intervention of lytic phages. In the study of Papaianni et al. (2020) [15] the lytic activity of Xccφ1 (*Xanthomonas campestris* pv. *campestris*-specific phage) was evaluated in combination with 6-pentyl-α-pyrone (a secondary metabolite produced by *Trichoderma atroviride* P1) associated with hydroxyapatite. The results demonstrated that Xccφ1, alone or in combination with 6PP and HA (φHA6PP complex), interferes with the gene pathways involved in the formation of biofilm, by modulating the genes involved in the biofilm formation and stability (*rpf*, *gumB*, *clp* and *manA*). This approach can be used as a model to fight against other biofilm-producing bacteria.

With the hypothesis that the use of antimicrobial textiles may significantly reduce the risk of nosocomial infections, Hamed et al. (2020) [16] focused their attention on endophytic fungi isolated from marine organisms, collected from saline environments. The antimicrobial and antioxidant activities of 32 fungal isolates were examined against a panel of pathogenic bacteria and fungi. The ethyl acetate crude extracts of 21 strains initially possessed antimicrobial or antioxidant activities. As an innovation, the surface of cellulosic fabrics was functionalized by grafting of MCT-βCD (monochlorotriazinyl β-cyclodextrin), creating core-shaped hydrophobic cavities. Furthermore, inclusion of the three most active fungal extracts (*Aspergillus calidoustus* M113, *Aspergillus terreus 7S4, Alternaria alternata 13A*) into the hydrophobic cavities was achieved. The experience demonstrated that *Aspergillus calidoustus* strain M113 exhibited the most promising improvement in the antimicrobial functionality, and the second best in the UV protection of this novel generation of fabrics. A low/weak toxicity against normal human skin fibroblasts was determined. Large-scale production of this bioactive extracts as well as the industrial application of the process to develop eco-friendly multifunctional textiles, is the first step to determine the economic feasibility.

Based on in silico techniques that have recently gained attention in drug discovery programs (e.g., structure and ligand-based virtual screening, docking and molecular dynamics), the team of Sayed (2020) worked on the protein Mpro [17]. This important protease of the severe acute respiratory syndrome coronavirus 2 (SARS-CoV-2) is a key of the viral infection, ensuring the cleavage of the two replicase proteins pp1a and pp1ab. Mpro was subjected to hyphenated pharmacophoric-based and structural-based virtual screenings. The Natural Products Atlas (N. P. Atlas), a data base of more than 24,000 microbial natural compounds, was screened to find out analogues exhibiting antiviral activity, by adapting to the catalytic site of Mpro. Using Lipinski’s rules, 9933 drug-like candidates were detected. Top-scoring hits were further filtered out depending on their ability to show appropriate binding affinities towards the molecular dynamic simulation (MDS)-derived enzyme’s conformers. Six compounds exhibiting high potential as anti-SARS-CoV-2 drug candidates were consequently detected. Further in vitro testing of the selected molecules is a promising starting point for the rapid development of medicament candidates against SARS-CoV-2.

## 5. Challenges in Production Steps

Omic sciences now make it possible to refine our knowledge on the biological transformations underlying biotechnologies.

The wine industry is making significant progress thanks to research carried out on the yeast *Saccharomyces cerevisiae*. The work of Gonzáles-Jiménez et al. (2020) [18] makes it possible to draw a proteomic map in order to distinguish the protein content of sparkling wines produced by different strains of *Saccharomyces cerevisiae*. The study shows that the proteins in the yeasts, that are responsible for the production of the volatile compounds released during sparkling wine elaboration, are quite similar in a flor yeast and a conventional one, except for the proteins Adh1p, Fba1p, Tdh1p, Tdh2p, Tdh3p, and Pgk1p. These proteins are present in higher concentrations in the flor yeast versus the conventional strain. The higher concentration of these proteins may explain the different organoleptic properties obtained when ageing using flor yeasts. The mannoproteins released as well as compounds derived from autolysis and enzymes are involved in reactions that affect some aroma precursors and thus the specific volatilome. Proteomic analysis can thus be the prerequisite for a more precise characterization of the specific quality of a wine.

Genetic modifications today provide science with incredible tools to promote the production of molecules of interest in host organisms other than the original producers. The use of bacteria as productive hosts makes it possible to no longer depend on the geoclimatic and spatial constraints, inherent in plant cultures. Moreover, the molecules can be produced in low-cost media at relatively high growth rates, allowing the utilization of sunlight as an energy source for sustainable cultivation and production processes. In the work of Hilgers et al. (2020) [19], the heterologous host *Rhodobacter capsulatus* was used to improve the plant sesquiterpenoid pathway leading to β-caryophyllene. A maximum production of 139 ± 31 mg·L^−1^ was obtained. As the sesquiterpene usually demonstrates beneficial anti-phytopathogenic capacities, the bioactivity of β-caryophyllene and its oxygenated derivative was determined against diverse phytopathogenic fungi. The molecules significantly inhibited the growth of the 2 phytopathogenic *Sclerotinia sclerotiorum* and *Fusarium oxysporum*, while other were left unaffected, including some plant growth-promoting bacteria. Thus, the production of β-caryophyllene and β-caryophyllene oxide in *Rhodobacter capsulatus* can be considered as promising process for the production of natural compounds for the management of some plant pathogenic fungi in agricultural crop production.

The safe production of amino acids for the food and feed industry has already been widely established with *Corynebacterium glutamicum* as the major production host. The challenge of the work of Walter et al. (2020) [20] was to favor a specific pathway for the production of a N-functionalized amino acid N-methylanthranilate (NMA). This amino-acid is a major precursor of bioactive compounds such as anticancer acridone alkaloids and other peptide-based drugs. However, the current routes of chemical synthesis or biosynthesis of N-alkylated amino acids cannot be easily exploited, due to insufficient yields or low profitability. The research work develops a fermentative NMA production by metabolic engineering of genome-reduced chassis strain *C. glutamicum C*1 combined with the introduction of a SAM-dependent ANMT (N-methyl transferase enzyme) gene, originating from *Ruta graveloens*. With this engineered strain the production of NMA reached a final titer of 0.5 g·L^−1^ with a yield of 4.8 mg·g^−1^ glucose, which has never been obtained before. Very specific transformations adapted to market needs are thus possible. 

After strain characterization or improvement and metabolite production, the extraction step is a key step toward the industrial production. A microbial product’s extraction is often solvent consuming and inconsistent with the principle of sustainable environment. In the study of Lebeau et al. (2020) [21], the azaphilone red pigments and ergosterol derivatives produced by a wild type of marine derived *Talaromyces* species are described. Alternative extraction process of the fungal pigments is developed, using a high pressure with eco-friendly solvents. These fungal pigments could be of interest due to their applications in the design of new pharmaceutical products.

However, Herculean work must be undertaken for the optimization of the molecules’ extraction. It would be pointless to promote the production and use of microbial natural molecules, while simultaneously releasing a greater quantity of pollutants into ecosystems.

## 6. Improving the Biotechnological Process and Opening to Unusual Fields

Improving the biotechnological processes is one of the cornerstones of the transition from pilot-scale fermenters to industrial applications. 

Jawan et al. (2020) [22] aim to optimize the growth parameters of *Lactococcus lactis* Gh1 in regard with the production of BLIS (bacteriocin-like inhibitory substances). Indeed, lactic acid bacteria (LAB) bacteriocins are considered good bio-preservative agents due to their non-toxic, non-immunogenic and thermo-resistance characteristics as well as broad bactericidal activity. The large-scale production of BLIS can offer promising opportunities for the development of efficient food bio preservation strategies. The parameters such as the age and size of the inoculum, initial pH of culture media and the nature of nitrogen and carbon sources, were studied in the study. Thanks to this work, the best set of parameters was defined, based on the production of BLIS. It can be concluded that bacteriocin production can be notably improved by altering the cultivation conditions of the bacterium. Results could be used for subsequent application in process design and optimization at larger scale.

Oslan et al. (2020) [23] worked on improving the growth and viability of the mutant *gdhA Pasteurella multocida* B:2. As they have demonstrated that *gdhA P. multocida* B:2 growth was inhibited by the accumulation of by-product ammonium in the culture medium, they developed a 2 L stirred-tank bioreactor integrated with an internal column using cation-exchange adsorption resin. This way, they demonstrated a significant improvement in growth performance and viability of the bacteria, by continuously reducing the in situ inhibitory effect of ammonium.

Efficiently increasing the yields of specific metabolites undeniably requires the use of computer and statistical tools. 

The work of Venkatachalam et al. (2020) [24] fully involves computational tools to optimize the physico-chemical parameters (initial pH, temperature, agitation speed and fermentation time), for the production of pigments and biomass in submerged fermentation by a marine-derived strain of *Talaromyces albobiverticillius*. To achieve the optimization, a Box–Behnken experimental design (BBED) based on a three-interlocking, 22 factorial matrix was applied, reducing notably the number of experiments required. The results were statistically analyzed, and a prediction of the optimal conditions was proposed using a response surface modeling (RSM) process. This methodology allowed easier consideration of multifactorial interactions between a set of diverse parameters and enabled a rapid selection of the best culture conditions, in regard with the various objectives set. Therefore, the predictive model was validated and the optimal conditions for the maximum production of pigments (red and orange) as well as biomass, were determined as follows: initial pH 6.4, 24 °C, agitation speed of 164 rpm for a fermentation time of 149h. This methodology greatly facilitates and accelerates the selection of the best experimental conditions for the production of metabolites of interest.

In the field of environment depuration, sugarcane distillery spent wash (DSW) is one of the most polluting industrial effluents. Its acidic pH, high mineral matters and chemical oxygen demand (COD) are involved in deep environmental disruptions. High added-value products generated from waste are particularly encouraged through the European directive 2009/28/CE [3] and are an economically favorable way to perform bioremediation. Thus, Chuppa-Tostain et al. (2020) [25] selected 37 strains of yeasts and filamentous fungi to test their abilities in reducing the organic load of vinasses, through measuring their impact on COD, pH, minerals and OD_475nm_ parameters. Among the strains tested, the species from *Aspergillus* and *Trametes* genus offered the best results in the depollution of DSW. The increase in soil and water pollution will certainly require, in the coming years, a concentration in research efforts in the field of biological purification.

The agro-food-processing industrial effluents generated in the maize (*Zea mays*) cooking process, are also considered as environmental pollutants. Some scientific programs aim at studying the utilization of these wastewater as raw substrate for microorganism’s growth and bioactive metabolite production. The research of Bacame-Valenzuela et al. (2020) [26] focused on the culture conditions for the production of pyocyanin (a redox metabolite of the phenazine group) by *Pseudomonas aeruginosa*. The parameters were first optimized using statistical design and RSM (response surface methodology) in a defined medium. The optimized parameters were then applied in a culture using an alkalinized maize-based effluent. The production of pyocyanin was up to 3.25 μg·mL^−1^, higher than in initial defined medium. In this way, valorization of lime-cooked maize wastewater used as a substrate for microbial growth was demonstrated in the production of a value-added compound.

In a complementary way, Zhang et al. [27] focused on the process for lactic acid production from corn stover, a common agricultural residue. Lignocellulose residues are one of the most abundant renewable feedstocks. They selected a *Pediococcus acidilactici* safe strain (PA204) with a high-efficiency for the utilization of xylose, a good ability to ferment sugar derived from lignocellulose, and a high temperature tolerance. To improve the lactic acid production, they developed a fed-batch simultaneous saccharification and fermentation (SSF) process (at 37 °C, pH 6.0) using corn stover and corn steep powder as carbon sources, in a 5 L bioreactor. The culture produced up to 104.11 g·L^−1^ lactic acid and a yield up to of 0.77 g·g^−1^ in regard with the NaOH-pretreatment applied on corn stover and the addition of cellulases. This study developed a feasible and efficient fed-batch process for lactic acid production from corn stover, and provides a promising candidate strain for high-titer and -yield lignocellulose-derived lactic acid production.

## 7. Future Strategies and Perspectives

Due to technical and scientific progress, the future is rich with new opportunities to go further in the use of microorganisms as controlled cell factories, producing high value-added metabolites for our daily needs.

As an example, even if filamentous actinobacteria are historically known and used as antibiotic producers, novel approaches are on the way. Protoplast fusion is an ancient technique for genome recombination that drastically increases microbial genetic diversity and favors the expression of silent or poorly expressed genes. However, the method requires multiple fusion and regeneration phases. Moreover, cells face a short time frame for recombination, a consequence of the necessity for protoplasts to regenerate their cell wall, and additionally, the recombinants are commonly fickle. These disadvantages are clear limitations for the production and the exploitation of original valuable metabolites. The article of Shitut et al. (2020) [28] highlights the potential of new engineered wall-deficient bacteria having the capacity to proliferate without their cell wall. Unlike protoplasts, L-forms are able to stabilize multiple chromosomes over many cycles of division. After the fusion, the period of recombination would thus be lengthened. Gene expression could also be based on the two parental genomes. These advances would significantly contribute to increase the chemical diversity of molecules produced by the engineered cells. These new L-forms open a perspective for the discovery of novel compounds, especially in the field of antibiotic research.

Cyanobacteria, as well as all photosynthetic organisms, have the capacity to produce secondary metabolites based on solar energy and carbon dioxide utilization, fitting a current sustainable development philosophy. Metabolic engineered-based approaches are now widely applied to cyanobacteria for the industrial production of value-added compounds, including specific metabolites and non-natural biochemicals. However, the review of Jeong et al. (2020) [4] covers a large synthetic and systems biology approach for advanced metabolic engineering of cyanobacteria. This involves an overview of known biosynthetic clusters coding for essential compounds, and knowledge of heterologous expression for cyanobacterial secondary metabolite production. As a perspective, the review highlights the development of next-generation sequencing (NGS) techniques, combined with the collection of omics data such as transcriptome, translatome, proteome, metabolome or interactome, which enrich the worldwide databases, opening huge opportunities to better manipulate and control cyanobacteria productions. The development of an in silico model at the genome scale (GEM) could quickly make it possible to remedy the current limitations of cyanobacterial engineering.

As a conclusion, we are very happy that we received 25 reviews/perspectives/original papers for publication in this Special Issue. We wish to thank all the authors and reviewers for their significant contributions and for making it a highly successful and timely collection of studies.
